# Restoration of p53 using the novel MDM2-p53 antagonist APG115 suppresses dedifferentiated papillary thyroid cancer cells

**DOI:** 10.18632/oncotarget.17398

**Published:** 2017-04-24

**Authors:** Haibo Chen, Dingyuan Luo, Lin Zhang, Xiaofeng Lin, Qiuyun Luo, Hanjie Yi, Jing Wang, Xianglei Yan, Baoxia Li, Yuelei Chen, Xingguang Liu, Hong Zhang, Sheng Liu, Miaozhen Qiu, Dajun Yang, Ningyi Jiang

**Affiliations:** ^1^ Department of Nuclear Medicine, Sun Yat-Sen Memorial Hospital, Sun Yat-Sen University, Guangzhou 510120, China; ^2^ Department of Vascular and Thyroid Surgery, Sun Yat-Sen Memorial Hospital, Sun Yat-Sen University, Guangzhou 510120, China; ^3^ Department of Experimental Research, State Key Laboratory of Oncology in South China, Collaborative Innovation Center for Cancer Medicine, Sun Yat-Sen University Cancer Center, Guangzhou 510060, China; ^4^ Department of Clinical Laboratory, Sun Yat-Sen University Cancer Center, Guangzhou 510060, China; ^5^ Department of Pharmacy, The First Affiliated Hospital of Sun Yat-Sen University, Guangzhou 510080, China; ^6^ The State Key Laboratory of Cell Biology, Institute of Biochemistry and Cell Biology, Shanghai Institutes for Biological Sciences, Chinese Academy of Sciences, Shanghai 200031, China; ^7^ Department of Medical Oncology, Sun Yat-Sen University Cancer Center, Guangzhou 510120, China; ^8^ Suzhou Ascentage Pharma Inc., Jiangsu 215123, China

**Keywords:** dedifferentiated thyroid cancer, radioiodine, anti-tumor, MDM2-p53 interaction, apoptosis

## Abstract

Dedifferentiated papillary thyroid cancer (DePTC) is characterized by aggressive growth, recurrence, distant metastasis, and resistance to radioactive iodine (RAI) therapy. DePTC is also accompanied by poor prognosis and high early-mortality. Nevertheless, most DePTC cells show intact p53 downstream functionality. In cells with wild-type p53, the murine double minute2 (MDM2) protein interacts with p53 and abrogates its activity. Inhibition of the MDM2-p53 interaction restores p53 activity and leads to cell cycle arrest and apoptosis. Restoring p53 function by inhibiting its interaction with p53 suppressors such as MDM2 is thus a promising therapeutic strategy for the treatment of DePTC. The novel MDM2-p53 interaction antagonist APG115 is an analogue of SAR405838, and is being tested in a phase I clinical trial. In this study, we evaluated the efficacy of APG115 as a single-agent to treat DePTC. APG115 diminished the viability of p53 wild-type DePTC cells and induced cell cycle arrest and apoptosis. In a human xenograft mouse model, APG115 elicited robust tumor regression and cell apoptosis. These data demonstrate that further research is warranted to determine whether APG115 can be used to effectively treat DePTC patients.

## INTRODUCTION

Thyroid carcinoma is the most prevalent subtype of endocrine malignant neoplasm [1]. Differentiated thyroid cancer (DTC) consists mostly of thyroid carcinoma (more than 90% of cases), and is classified as either papillary (PTC, 75-80%) or follicular thyroid carcinoma (FTC, 5-10%) [1–3]. In 2015, ~900,00 new cases of thyroid carcinoma were diagnosed in China, resulting in approximately 6,800 deaths [4]. The rapidly rising incidence seen in recent decades in both China and the United States (US) [4, 5] can be attributed to improved accuracy in the detection of papillary thyroid cancer (PTC) [6]. Patients with DTC, especially PTC, generally have good prognosis and can be cured by traditional and radioactive iodine (RAI) therapies [1, 2]. Nevertheless, approximately 5-15% of the cases are dedifferentiated papillary thyroid cancer (DePTC), which is associated with poor prognosis and high early-mortality [7–9]. Cellular dedifferentiation generally occurs in cases accompanied by more aggressive growth, recurrence, distant metastasis, and resistance to RAI [1, 8–10].

Few effective treatment strategies are available for patients with DePTC. External-beam radiotherapy (EBRT) can rarely be administrated [10]. Furthermore, although doxorubicin is the only FDA-approved anti-tumor drug to treat thyroid cancer, its therapeutic efficacy is discouraging due to the low response rate and poor survival rates of patients who take it [11]. Only two multi-targeted tyrosine kinase inhibitors (TKIs), sorafenib and lenvatinib, have been approved by the Food and Drug Administration (FDA) in the US to treat RAI-refractory metastatic DTC, which has also changed the standard clinical approach to treat DePTC [12, 13]. Although these two multi-targeted TKIs improve patient response in terms of progression free survival (PFS), the data showing improvement in overall survival are very limited [14]. Moreover, these drugs might induce severe negative side effects resulting in treatment interruption [1]. Consequently, further efforts are needed to develop new drugs that can more effectively treat patients with DePTC.

A newly developed tactic is to target the hydrophobic protein–protein binding site between murine double minute-2 (MDM2) and its transcriptional factor p53. The tumor suppressor p53 can regulate many biological processes such as cell cycle arrest, DNA damage, cellular senescence, and apoptosis [15, 16]. p53 acts as a transcription factor for MDM2 through a negative feedback loop, and MDM2 in turn acts as an E3 ubiquitin ligase targeting p53 for proteasomal degradation. Overexpression of MDM2 disrupts the well-established balance between MDM2 and p53, resulting in tumor initiation [17]. Accumulated MDM2 levels can be obtained from MDM2 gene amplification or by other mechanisms [18]. Targeting and blocking the hydrophobic MDM2-p53 binding site might therefore restore the normal anti-tumor effect of p53.

Indeed, using MDM2-p53 interaction antagonists has recently become an effective treatment for various preclinical cancer models such as soft tissue sarcoma, acute leukemia, colon cancer, prostate cancer, adenoid cystic cancer [19], neuroblastoma [15, 20, 21], and lung cancer [17, 22]. Such treatment can restore wild-type p53 and elicit p53-mediated cancer cell death. Furthermore, multiple MDM2-p53 interaction antagonists, such as the nutlin analogue RG7112, SAR405838, and its derivative APG115, are being used in clinical trials [23, 24]. However, Phase I trial results suggest poor tolerability to daily oral administration and that thrombocytopenia is a dose-limiting factor [15]. An additional defect of SAR405838 and its previous analogues is that they slowly racemize in solution [25, 26]. Therefore, MDM2-p53 interaction antagonists with enhanced specificity and efficacy are needed to restore p53 activation at lower concentrations.

To date, less than 10% of papillary thyroid carcinomas harbor p53 mutations [27]. Accordingly, many patients with DePTC might harbor wild-type p53. Furthermore, nuclear accumulation of MDM2 protein might promote pathogenesis in a subset of PTC [28]. Hence, restoration of normal p53 levels using MDM2-p53 interaction antagonists may be a feasible strategy for DePTC treatment. In the present study, we investigated the anti-tumor efficacy of APG115 and the associated underlying mechanisms in DePTC cell lines both *in vitro* and *in vivo*.

## RESULTS

### APG115 inhibits DePTC proliferation in a p53-dependent manner

**Figure 1:**
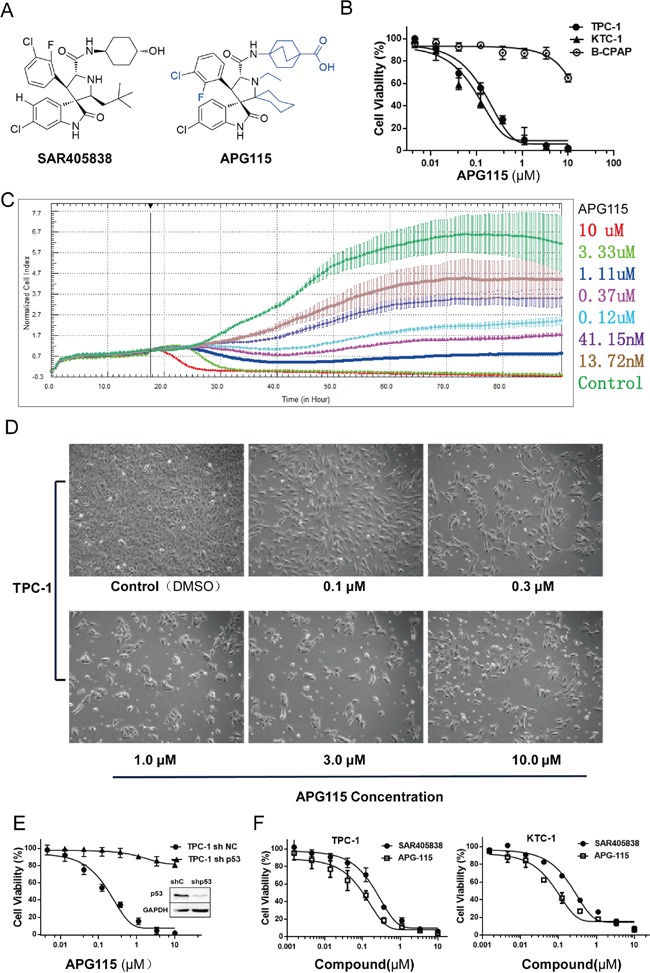
The novel MDM2-p53 interaction antagonists APG115 and its analogue inhibited p53 wild-type DePTC cells growth **(A)** The structure of novel MDM2-p53 interaction antagonist APG115 and its analogue SAR405838. **(B)** APG115 inhibited wild-type p53 DePTC cells proliferation in a concentration-dependent manner but not in mutated p53 DePTC cells (B-CPAP). **(C)** Cell proliferation Kinetics was measured by continuous time-lapse cell imaging using the xCELLigence RTCA system. **(D)** TPC-1 cells morphology profile changed in response to incubation with the indicated concentrations of APG115 for 72 h. **(E)** APG115 inhibited the proliferation of DePTC cells in a p53-dependent manner. Cell viability was unaffected by APG115 following stable p53 knockdown in TPC-1 cells compared with non-target controls. **(F)** MTS assays measured cell viability of wild-type p53 DePTC cell lines after incubating with increasing concentrations of APG115 and its analogue SAR405838 for 72 h.

The structure of the novel MDM2-p53 interaction antagonist APG115 and structural differences compared to its analogue SAR405838 are shown in Figure 1A. We found that APG115 inhibited cell proliferation in wild-type p53 DePTC cell lines (TPC-1 and KTC-1) in a concentration-dependent manner over a 72-hour period, but to a much lesser extent in the p53-mutated DePTC cell line (B-CPAP) (*P* < 0.0001). The DePTC cell lines with wild-type p53 had nanomolar IC_50_ values of 133.4 ± 28.3 nM (mean±standard deviation (SD)) for TPC-1, and 94.8 ± 38.0 nM (mean ± SD) for KTC-1). On the other hand, the p53-mutated DePTC cell line had an IC_50_ value of 77.8 ± 22.5 μM (mean ± SD) (Figure 1B) (Supplementary Table 1). APG115 inhibited TPC-1 cells (wild-type p53) growth in a concentration-dependent manner as measured by the xCELLigence real-time cell analysis (RTCA) system (Figure 1C) and cell morphology profiles (Figure 1D, Supplementary Figure 1). Additionally, cell growth kinetics and change of morphology illustrated that the onset of cell death was relatively slow, with visual signs of adhesion loss in response to APG115 treatment at doses greater than 300 nM in DePTC cells retaining wild-type p53.

To further validate whether the anti-proliferative effect of APG115 was strictly dependent on the status of functional p53, we stably knocked down p53 by short hairpin interfering RNA (shRNAi). TPC-1 p53 knocked-down (TPC-1 sh-p53) cells and TPC-1 p53 knocked-down negative control (TPC-1 sh-NC) cells were treated with increasing concentrations of APG115 (serially diluted 1:3 and run in a concentration series from 0 to 10 μM). Cell viability was unaffected by APG115 treatment following stable p53 knockdown compared with stably transfected negative controls (*P* < 0.0001; Figure 1E). The IC_50_ value for stably transfected negative control cell line TPC-1 sh-NC was 158.2 ± 30.3 nM (mean ± SD), whereas the IC_50_ value for stable p53 knockdown cell line TPC-1 sh-p53 was 445.6 ± 49.2 μM (mean ± SD) (Supplementary Table 1).

In addition, APG115 was approximately three times more potent than SAR405838 in decreasing the viability of TCP-1 cells (*P* < 0.01) and KTC-1 cells (*P* < 0.01, Figure 1F). The IC_50_ values of SAR405838 were 576.3 ± 17.5 nM and 276.6 ± 42.3 nM (mean ± SD) for TPC-1cells and KTC-1 cells, respectively (Supplementary Table 1).

### APG115 induces cell-cycle arrest and apoptosis in a p53-dependent manner

**Figure 2:**
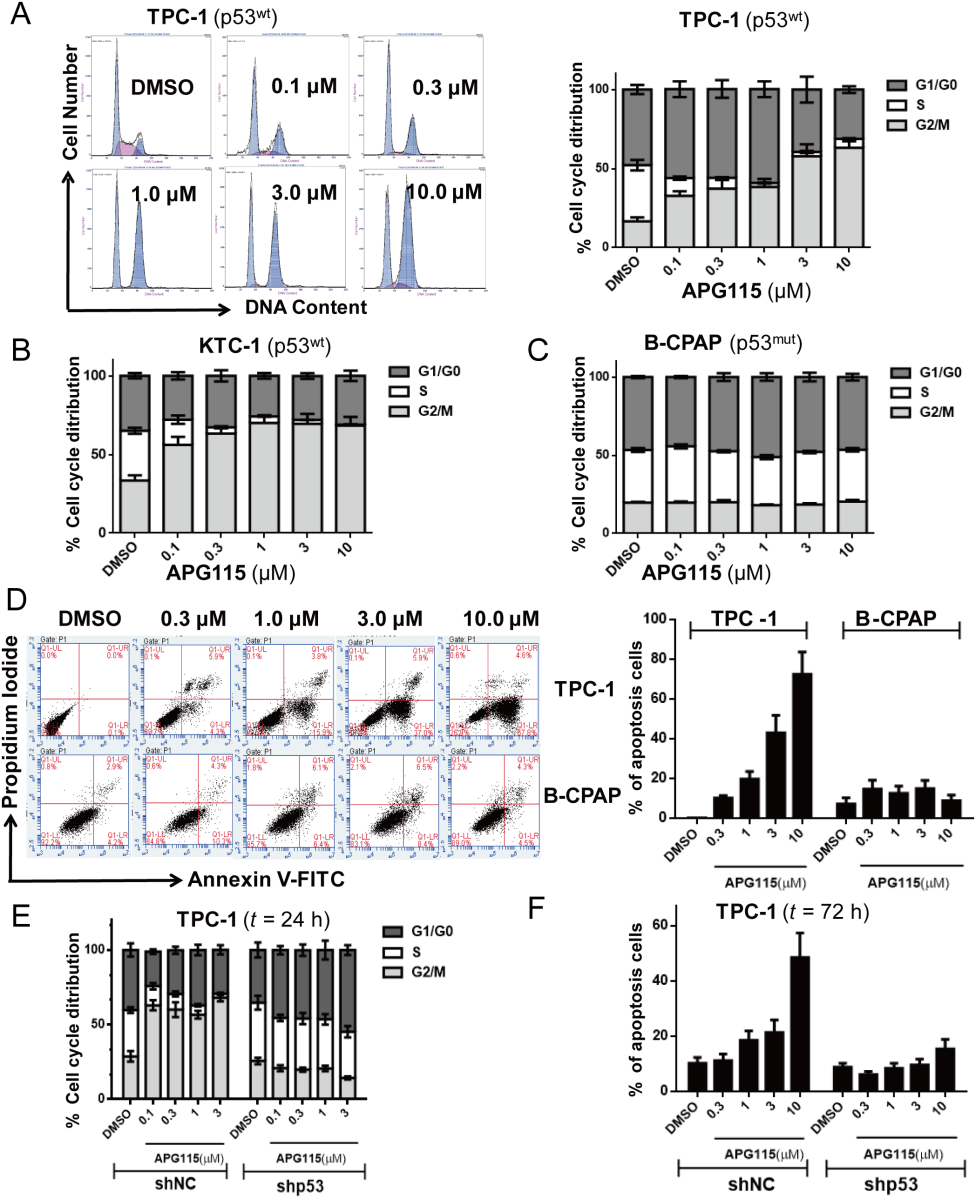
APG115 elicited cell cycle arrest and apoptosis in a p53-dependent manner in DePTC cells **(A-C)** DePTC cells were incubated with the indicated concentrations of APG115 for 24 h. The cell cycle was detected by flow cytometry. APG115 induced a concentration-dependent cell cycle arrest in G2/M phases and a reduction in the number of cells in S-phase in TPC-1 and KTC-1 cells retaining wild-type p53, but not in B-CPAP cells with mutated p53. **(D)** DePTC cells were treated with the indicated concentrations of APG115 for 72 h and apoptosis was measured by flow cytometry. APG115 elicited a significant concentration-dependent increase in apoptosis in TPC-1 cells but not in B-CPAP cells. **(E-F)** Stable knockdown of p53 effectively abrogated cell cycle arrest and apoptosis induced by APG115 in TPC-1 cells.

Treatment of exponentially proliferating DePTC p53 wide-type cell lines (TPC-1, KTC-1) with APG115 for 24 h led to a concentration-dependent cell cycle arrest in G2/M phases and a decrease in the number of cells in S-phase. In response to increasing concentrations of APG115 (0-10 μM), the TPC-1 cell population in S-phase reduced from 35.4% to 2%, whereas accumulation of cells at G2/M phases increased from 16.7% to 63.2% (Figure 2A). The same effect was seen in the KTC-1 cell line, with a decreasing of the S-phase population from 31.7% to 0.6% (Figure 2B, Supplementary Figure 2). Nevertheless, this effect was not observed in the p53-mutated cell line B-CPAP (Figure 2C, Supplementary Figure 2).

In TPC-1 cells, the apoptosis rate was increased in a concentration-dependent manner after 72 h treatment with DMSO, 0.3, 1, 3, 10 μM of APG115, respectively. The dose of APG115 required to achieve 50% of the maximum reduction of the S-phase population (EC_50_: 0.08 μM) was approximately seven-fold lower than the dose required to induce apoptosis (EC_50_: >3 μM) in the TPC-1 cell line. However, APG115 led to only 1.5-fold increase in cell apoptosis at 10 μM in the B-CPAP cell line (Figure 2D). Stable knockdown of p53 in the TPC-1 cell line effectively abrogated cell cycle arrest (Figure 2E) and apoptosis (Figure 2F) in response to APG115 treatment. The cell cycle and apoptosis were unaffected by APG115 in TPC-1 sh-NC (*P* < 0.0001).

### APG115 stabilizes p53 and induces an increase in the expression of p53 downstream targets in p53 wild-type DePTC cells

**Figure 3:**
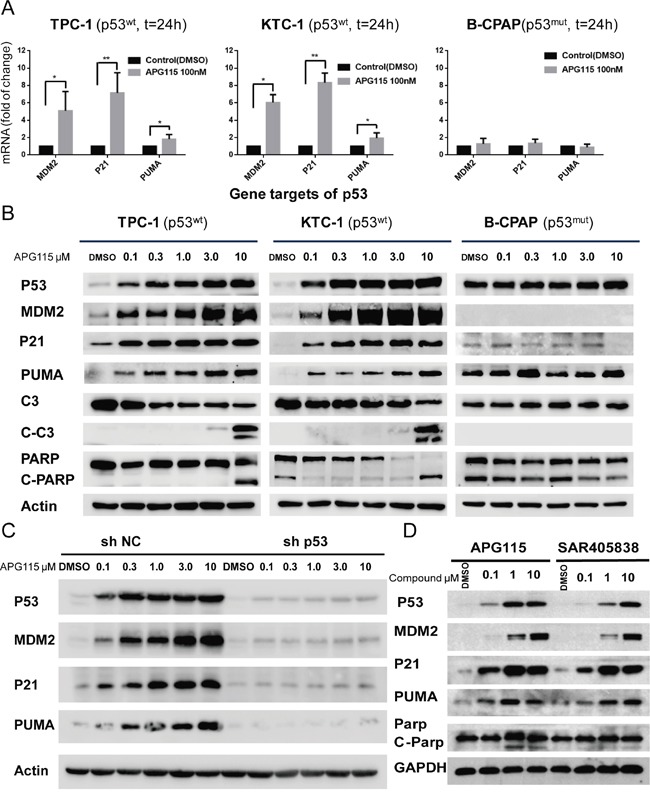
APG115 restores p53 downstream signaling pathway, and elicits apoptosis in p53 wild-type DePTC cells **(A)** APG115 treatment for 24 h, upregulated the expression of p53 transcriptional targets mRNA in two wild type p53 DePTC cell lines, measured by quantitative PCR (*P<0.05, **P<0.01). No upregulation is seen in B-CPAP cells with mutated p53. **(B)** Western blot analysis evaluating the effects of 24 h incubation with increasing concentrations of APG115 in three DePTC lines. APG115 elicited restoration of p53, upregulation of MDM2 protein expression, and subsequently activated p53-mediated cell cycle and apoptosis and an increase in the expression of p21, Puma, cleaved Caspase- 3(C-C3), and cleaved-PARP(C-PARP) proteins. **(C)** The stable knockdown of p53 significantly abrogated the expression of p53 transcriptional target genes following MDM2-p53 interaction blocking. **(D)** TPC-1 cells were incubated with the indicated concentrations of APG115 and its analogue SAR405838 for 24 h. The protein expression of p53 and its target genes was analyzed by western blotting.

APG115 led to a rebound upregulation of the mRNA expression of p53 transcriptional targets MDM2 (involved in forming a feedback loop with p53), P21 (pan-cyclin–dependent kinase inhibitor), and PUMA (a marker involved in p53 apoptotic activity). APG115 induced a 5-fold increase in MDM2 expression (*P* =0.0165), a 7-fold increase in p21 expression (*P* =0.0054), and a 2-fold increase in PUMA expression (*P* =0.0342) in TPC-1 cells. A similar increase of the same genes was detected in response to APG115 treatment in KTC-1 cells. Nevertheless, there was no upregulation in cell line B-CPAP expressing mutant p53 (Figure 3A).

Increasing concentrations of APG115 resulted in a concentration-dependent accumulation of p53 protein with subsequent restoration of transcriptional targets involved in cell-cycle arrest and apoptotic stress responses. This effect was detected in two DePTC cell lines with wild type p53, whereas there was no increase in p53 and its transcriptional targets in B-CPAP cells (Figure 3B). Furthermore, efficient stable knockdown of p53 in TPC-1 (TPC-1 sh-p53) cells effectively abrogated expression of p53, p21, MDM2, and PUMA (Figure 3C) in response to APG115 treatment, whereas stably transfected negative control shRNAi TPC-1 (TPC-1 sh-NC) cells did not cause concentration-dependent upregulation of those proteins.

To validate whether the novel MDM2-p53 antagonist APG115 is more potent than its analogue SAR405838 in restoring p53 activity, TPC-1 cells were incubated with increasing concentrations of APG115 or SAR405838 for 24 h. Both of the two antagonist restored p53 activity in a dose-dependent manner. However, APG115 was approximately three times more potent than SAR405838 in restoring p53 protein levels (Figure 3D).

### APG115 elicits strong anti-tumor responses in DePTC xenograft models

**Figure 4:**
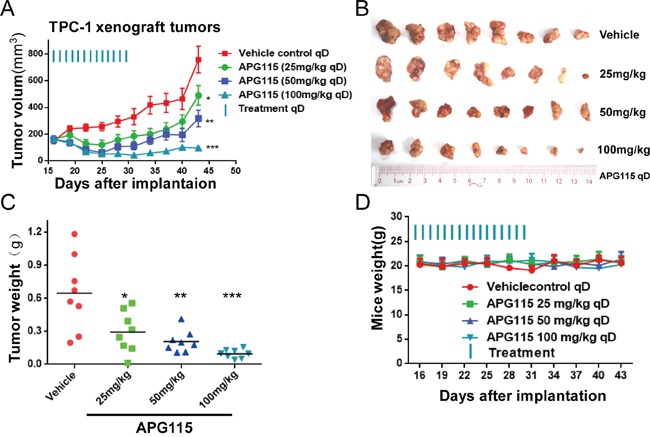
APG115 induced tumor regression in a TPC-1 xenograft tumor model **(A)** Graph depicting average tumor volume at the indicated time in BALB/c nude mice receiving 25, 50, 100 mg/kg of APG115 or vehicle via daily oral gavage for 15 days. Treatment started when the volume of the tumors was 150~200 mm^3^. The data are plotted as mean ± SD (n = 8/group). *, *P* < 0.05; **, *P* < 0.01; ***, *P* < 0.001. **(B)** Photographs of the xenograft tumors retrieved immediately at the end of the experiment. **(C)** Graphs depicting the weight of tumors resected immediately from mice after euthanasia. The data are plotted as mean ± SD (n = 8/group). *, *P* < 0.05; **, *P* < 0.01; ***, *P* < 0.001. **(D)** Graph depicting mouse weight through the duration of the experiment.

We first evaluated the effect of APG115 *in vivo* DePTC xenografts. Daily oral administration of APG115 inhibited TPC-1 subcutaneous xenografts tumor growth in a dose-dependent manner. Compared to an average volume of 758 ± 110.2 mm^3^ for the control vehicle group, APG115 at daily doses of 25 mg/kg and 50 mg/kg for 15 days elicited tumor suppression of 35.4% and 58.2%, respectively (Figure 4A and 4B). At the high dose of 100 mg/kg, a majority of TPC-1 xenograft tumors regressed and the average tumor volume was below the primary average volume. The tumor regression obtained in response to APG115 treatment at 100 mg/kg was long-lasting; at day 46, 13 days after the last treatment, the average tumor volume of all eight mice remained below the primary volume. These data were also consistent with the evaluation of tumor weight at the end of the experiment (Figure 4C). APG115 was well tolerated in those mice, as demonstrated by the lack of obvious weight loss upon treatment (Figure 4D).

### APG115 restores the p53 signaling pathway and induces apoptosis in DePTC xenografts

**Figure 5:**
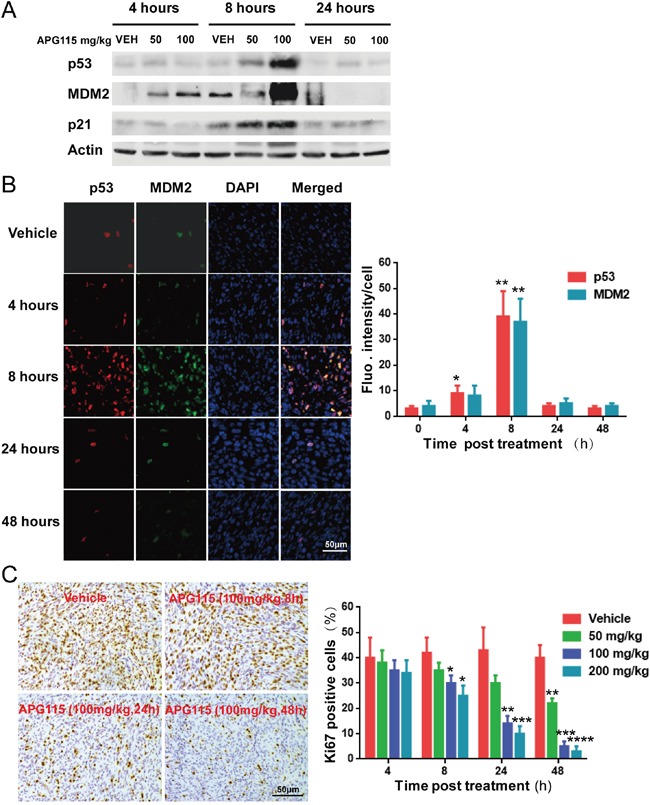
Pharmacodynamics of APG115 in TPC-1 bearing nude mice **(A)** Western blot assay showing the upregulation of p53, MDM2, and p21 protein expression in TPC-1 xenograft tumors. Lysates were prepared from tumors treated with vehicle, or with 50 or 100 mg/kg of APG115 (n=3/group). **(B)** Representative immunofluorescence images of TPC-1 bearing nude mice 4 to 48 h post APG115 treatment (single dose of 100 mg/kg) or control vehicle treatment. 53 (red), MDM2 (green) and cell nuclei (blue) can be seen. p53 and MDM2 levels increased in the presence of 100 mg/kg of APG115 4 h after treatment, peaking at 8 h, and returning to basal levels at 24 h (left panel). The photographs depict fluorescence intensity changes post treatment. Each bar represents fluorescence intensity per cell from 3 fields normalized over the value obtained in the corresponding untreated control mice (right panel). The data are plotted as mean ± SD. *, *P* < 0.05; **, *P* < 0.01. **(C)** Representative images of immunohistochemical detection of Ki-67 staining in TPC-1 xenograft tumors from control and APG115-treated mice (left panel). The bar graphs represent the mean ± SD of the number of Ki-67-positive cells from 3 microscopic fields in each group at multiple time points and APG115 doses (right panel). The data are plotted as mean ± SD. *, *P* < 0.05; **, *P* < 0.01; ***, *P* < 0.001, ****, *P* < 0.0001.

Western blot assay demonstrated a dose-dependent increase in p53 protein levels those of its targets MDM2 and p21 in athymic nude mouse bearing TPC-1 tumor tissues. The protein levels of p53 and its interactional protein MDM2 and p21 were highest at 8 h after treatment and returned to primary levels at 24 h (Figure 5A). We also found by immunofluorescence that p53 and MDM2 levels increased 4 h after treatment, peaked at 8 h, and returned to basal levels at 24 h in response to APG115 treatment (100 mg/kg)(Figure 5B).

**Figure 6:**
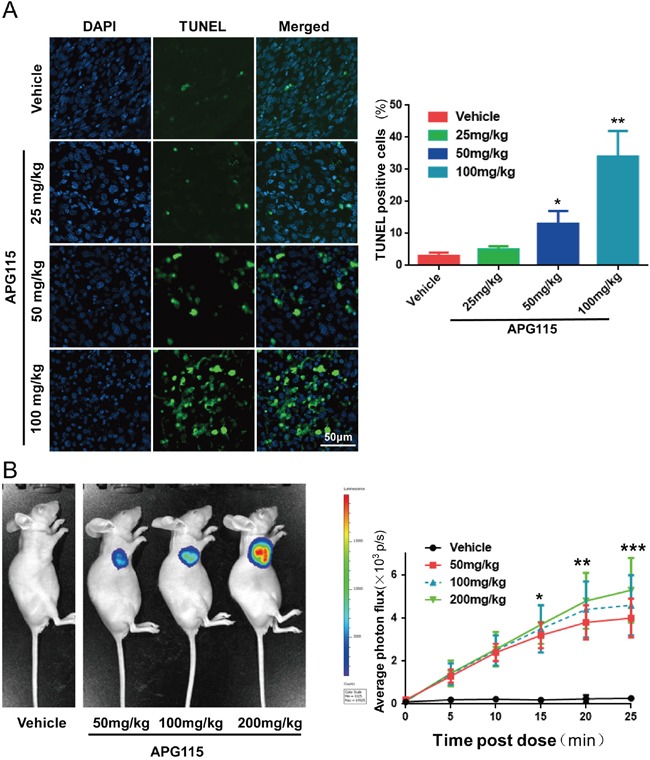
APG115 elicited TPC-1 xenograft tumor cells apoptosis **(A)** Representative photomicrographs of TUNEL-positive cells (green) and DAPI (blue) in xenograft tumors from mice at 48 h post treatment with a single dose of vehicle, or 25, 50 or 100 mg/kg of APG115 (left panel). Graph depicting the percentage of TUNEL-positive cells from 3 microscopic fields in each group at multiple doses of APG115 (right panel). The data are plotted as mean ± SD. *, *P* < 0.05; **, *P* < 0.01. **(B)** Representative bioluminescence images of activated caspase-3 and caspase-7 in nude mice bearing subcutaneous TPC-1 xenografts, treated with a single dose of vehicle, or 50, 100 or 200 mg/kg of APG115. At 48 h post treatment, the mice were given 50 mg/kg of zDEVD-aminoluciferin by intraperitoneal injection and images were captured 20 min later (left panel). We measured bioluminescent signal intensity in each group from 0 to 25 min post-zDEVD-aminoluciferin administration (right panel). The Z-DEVD-aminoluciferin signal is plotted as mean ± SEM (n = 3/group). *, *P* < 0.05; **, *P* < 0.01; ***, *P* < 0.001.

Activation of p53 in TPC-1 cells caused cell-cycle arrest, followed by apoptosis *in vitro* (Figure 3). Immunohistochemical experiments revealed reduced levels of the proliferation marker Ki67 in a time- and dose-dependent manner after APG115 treatment (Figure 5C). At the highest doses (100, 200 mg/kg), APG115 started to suppress proliferation at 8 h post treatment. At the highest dose (200 mg/kg), only 3.1±2.5% of cells stained Ki67 at 48 h post treatment versus 40.3±5.6% for the vehicle control, suggesting that APG115 can effectively arrest the cell-cycle and tumor cell proliferation. On the other hand, apoptosis was delayed, being minimal at less than 24 h, but prominent at the highest dose of APG115 after 48 h post treatment. *In situ* TUNEL staining further confirmed the delay in apoptosis induced by APG115 treatment (Figure 6A). Nude mice bearing TPC-1 subcutaneous xenograft tumors were treated with a single oral dose of vehicle, or 25, 50, and 100 mg/kg APG115 for 48 h. Cells staining positively for TUNEL demonstrated morphological features typical of apoptotic cells such as condensation and nuclear fragmentation (Figure 6A, left). A large increase in TUNEL positive cells was seen in tumors after administration of 100 mg/kg APG115 while only a mild increase was seen after administration of 50 mg/kg. On the other hand, the number of TUNEL positive cells after administration of 25 mg/kg APG115 was similar to that of vehicle-treated controls (Figure 6A, right).

To further assess the ability of APG115 to elicit apoptosis in nude mice bearing subcutaneous xenografts, we used bioluminescence to measure the levels of activated caspases 3/7 in TPC-1-luc tumor-bearing mice. We used Z-DEVD-aminoluciferin as a substrate for activated caspase-3 and 7. The intensity of the luciferase signal can inform on apoptosis levels in tumor tissues. Representative bioluminescent graphs from nude mice bearing vehicle and APG115 treated tumors are shown in Figure 6B (left). Xenograft tumors from nude mice reached approximately 200 mm^3^ in size when treated with a single concentration of 50, 100 or 200 mg/kg APG115. At 48 h post treatment, those tumors produced time- and concentration-dependent induction of luciferase signal as compared to untreated controls, demonstrating that apoptosis was occurring within the tumors (Figure 6B, right).

## DISCUSSION

DePTC, which accounts for approximately 5-10% of patients with PTC, leads to resistance to traditional therapeutic strategies and worsens prognosis [9, 10, 29]. RET/PTC rearrangements are found in 30-40% of cases, whereas a higher prevalence of BRAF mutations (up to 70%) has been observed in DePTC [9]. PTC patients with RET/PTC rearrangements [30–32] or the BRAF^V600E^ mutation [33, 34] exhibit cell dedifferentiation. Both the cell lines KTC-1 and TPC-1 harbor mutations (RET/PTC1 and BRAFV^600E^, respectively) that upregulate MAPK/ERK signaling transductions associated with promoting tumor migration and metastases [35]. Furthermore, dedifferentiation in thyroid carcinoma is characterized by a loss of thyroid specific markers and a subsequent gain of proliferative capacity [36, 37]. Dedifferentiated thyroid cell lines usually overexpress genes involved in general metabolism, cell cycle, and proliferation [36]. In addition, p53 mutations in localized PTC are infrequent [27]. Therefore, cell lines KTC-1 and TPC-1 retaining intact p53 functions are ideal models to test the anti-tumor effects of the novel MDM2-p53 interaction antagonist APG115.

The two FDA approved multi-targeted TKIs sorafenib and lenvatinib have limited efficacy in DePTC patients [12, 13]. In search for better compounds, we investigated the anti-tumor activity of APG115 in DePTC cells. Although the development of antagonists to the MDM2-p53 interaction is an area of intense research [23]. To our knowledge, MDM2-p53 interaction antagonist has not yet been investigated in thyroid carcinomas. Analysis of the crystal structure of the MDM2-p53 complex showed that the interaction between MDM2 and its transcriptional factor p53 was primarily mediated by three hydrophobic residues, namely Phe19, Trp23, and Leu26 of a p53 peptide and a small deep hydrophobic cleft in MDM2 [38]. Spiro-oxindoles invented by Wang's laboratory constitute is a new class of MDM2-p53 interaction antagonists that were developed using a structure-based de novo design strategy [39]. Structure-based optimization of Spiro-oxindoles (including SAR405838) were designed to capture bind the hydrophobic protein–protein binding site between p53 and MDM2. The cellular mechanism of p53 restoration by spiro-oxindoles agreed with *in vitro* biochemical binding assays demonstrating that these potent MDM2 antagonists are capable of inhibiting the MDM2-p53 interaction in cells [40]. They induce an increase in transcription and protein levels of p53-target genes [41]. SAR405838 has demonstrated potent single-agent efficacy in various cell lines and xenograft tumor models and is being investigated in a Phase I clinical trial [42].

The novel MDM2-p53 antagonist, APG115, is derived from SAR405838. It is a new spiro-oxindole with two identical substituents at the carbon-2 of the pyrrolidine core, with more chemically stability. Meanwhile, it was shown a very high affinity to MDM2 (K_i_<1 nM), and potent cellular activity [26]. This antagonist is currently investigated in a phase I trial in patients with advanced solid tumor or lymphoma (NCT02935907). In our study, APG115 played a highly selective, potent anti-tumor role in wild-type p53 DePTC cells both *in vitro* and *in vivo*. It also restored p53 and upregulated p53 transcriptional target genes in DePTC cells with wild-type p53. APG115 inhibited proliferation and viability at 72 h more potently than its analogue SAR405838. Furthermore, APG115 demonstrated a greater ability to restore p53 activity than SAR405838.

Restoration of p53 is known to block cell-cycle progression and induces apoptosis in the presence of MDM2-p53 interaction antagonists [43–45]. Presumably, p53 induces cell cycle arrest to protect cells from propagation of damaged DNA before it is repaired, whereas apoptosis eliminates cells with unrepairable damage [44]. Our data here showed that APG115 elicited both cell-cycle arrest and apoptosis in a concentration-dependent manner but manifested different kinetics for these two cellular processes, with an early onset for cell-cycle blockage and a late onset for apoptosis. Furthermore, the concentration of APG115 required to achieve 50% of the maximum reduction of the S-phase population was approximately seven-fold lower than the concentration required to elicit apoptosis in the p53 wild-type DePTC cell lines.

In this research, we investigated whether blocking the interaction between MDM2 and p53 would exert a similar effect in p53-mutated DePTC cells. We found that wild-type p53 was needed for APG115 to function. Furthermore, knocking down p53 abrogated the anti-proliferation effect of p53, as well as its ability to induce cell-cycle arrest and apoptosis after treatment with APG115. Hence, wild-type p53 was needed for APG115 to function.

Thyroid carcinomas are more prevalent among women. Therefore, we conducted our tests in female nude mice to more closely mimic epidemiological and clinical scenarios [4, 5]. APG115 demonstrated promising oral pharmacokinetics in athymic nude mice, achieving dose-dependent p53 restoration and upregulation of p53 downstream transcriptional targets in TPC-1 xenograft tumor tissues, thereby inhibiting proliferation and inducing apoptosis in a dose-dependent manner. We also showed that cell-cycle blockage preceded apoptosis induction *in vitro*. Furthermore, daily oral administration of APG115 to TPC-1 bearing nude mice for only 2 weeks led to a dose-dependent suppression of neoplasm growth and robust neoplasm regression in the highest concentration group. Noticeably, no neoplasm regrowth was observed when the therapy was withdrawn and xenograft tumors were observed for another 2 weeks. As demonstrated by the maintenance of mice weight throughout the experimental period, the neoplasm regression was not accompanied by notable signs of systemic toxicities. In other words, APG115 successfully penetrated human DePTC xenograft tumors and induced remarkable anti-tumor effects without notable side effects.

In conclusion, our study reports preclinical evidence that substantiates the therapeutic potential of APG115 *in vitro* and *in vivo*. APG115 effectively inhibited the proliferation of DePTC cells and restored p53 activity *in vitro* in a p53-dependent manner with higher potency than its analogue SAR405838. The robust tumor regression we observed in xenograft models of DePTC with wild-type p53 after APG115 treatment suggests that this MDM2-p53 interaction antagonist may be used as a therapeutic alternative to treat DePTC patients.

## MATERIALS AND METHODS

### Compound

APG115 was kindly provided by Ascentage Pharma Group Inc (Suzhou, China). The purity of APG115 was 99.4% in specification. SAR405838 was purchased from Selleck Chemicals (Houston, USA). For *in vitro* assays, all compound were dissolved in dimethylsulfoxide (DMSO; Sigma Aldrich, MO, USA) at a stock concentration of 20 mM, stored at -20°C. The final concentration of DMSO to dilute compounds in culture media did not exceed 0.1%.

### Human papillary DePTC cell lines

Cell lines KTC-1 and TPC-1 (harboring BRAF^V600E^ mutation and RET/PTC1 rearrangement, respectively) have wild type p53, whereas B-CPAP (harboring the BRAF^V600E^ mutation) is a p53 mutant [46, 47]. KTC-1 and B-CPAP cells were kindly provided by Stem Cell Bank, Chinese Academy of Sciences (Shanghai, China). TPC-1 cells were purchased from Cobioer Biosciences Co., LTD (Nanjing, China). Cell lines were authenticated by short tandem repeat (STR) DNA profiling, and matched with their respective STR profiles as shown in previously published studies, and/or in the European Collection for Cell Cultures (ECACC) and the German Collection of Microorganisms and Cell Cultures (DSMZ) databases at all loci tested in common [48]. All cell lines were passaged less than one month prior to banking and experimentation. Cells were incubated with RPMI 1640 supplemented with 10% fetal bovine serum (Gibico, CA, USA) and 1% non-essential amino acids (Invitrogen, CA, USA) at 37°C in a humidified atmosphere with 5% carbon dioxide.

### Cell proliferation/viability assays

Appropriate densities of cells were seeded into 96-wrell plates and cultured with different doses of APG115 for 72 h. Cell proliferation/viability was evaluated by MTS assay, using the CellTiter-96^®^ Aqueous Non-Radioactive Cell Proliferation Assay kit (Promega, Madison, WI, USA), according to the manufacturer's instructions. Absorbance was measured at 490 nm (Beckman Coulter, Miami, USA). The Reed-Muench method was used to calculate IC_50_ values [49]. Each data point was determined by triplicate assays. Cell growth kinetics were analyzed using xCELLigence RTCA DP instrument (ACEA Biosciences, Hangzhou, China). Data analysis was conducted using xCELLigence RTCA Control Unit and the preinstalled software. The detailed information of this method has been depicted in previous articles [50]. Cells (1500/well) were incubated in E-plate 16 with indicated concentrations of MDM2-p53 interaction antagonists or with a DMSO control. The proportional changes in impedance were monitored incessantly and presented as the cell index (CI). Change of CI values was monitored for ~72 h, with kinetic measurements taken every 15 min.

### Flow cytometry and cell morphology analysis

DePTC cells were cultured in 6-well plates with the indicated concentration of MDM2-p53 interaction antagonist APG115 or with a DMSO control for 24 or 72 h respectively as devised. Samples were detected utilizing a Gallios flow cytometer (Beckman Coulter, Miami, USA) for cell cycle assays and BD Accuri C6 flow cytometer (Becton Dickinson, San Jose, USA) for apoptosis assays. For cell-cycle detection utilized propidium iodide (PI) Detection kit (KeyGen BioTECH, Nanjing, China) following manufacturer's instructions. The cell-cycle raw data was reanalyzed by MutiCycle for windows software (Phoenix flow systems, San Diego, USA). Apoptosis was measured using the Apoptosis Detection kit (BD Pharmagen, San Diego, USA). The Accuri C6 software was utilized to quantitate the percentage of apoptosis cells.

As a parallel comparison of the cytotoxicity assay conducted prior to apoptosis assay, the DePTC cells were cultured in 6-well plates. The cell morphology was obtained through microscopy prior to apoptosis assay. The cells were visualized with an Olympus IX73 inverted fluorescence microscope (Olympus, Tokyo, Japan) after 72 h of incubation with the indicated concentrations of APG115 or DMSO control as for the apoptosis assays.

### RNA extraction and real-time quantitative RT-PCR (RT-qPCR) analysis

DePTC cells were either incubated with APG115 (100 nM for 24 h) or DMSO control. The total RNA was extracted from DePTC cell lines using TRIZOL reagent (Life Technologies, Grand Island, USA). _C_DNA was converted by the GoScript Reverse transcription (RT) System (Promega, Madison, USA) following the instructions from the manufacturer. To assess change of target genes expression, RT-qPCR was conducted using Gotaq PCR Master Mix (Promega, Madison, USA) on a CFX96 Real-Time System (Bio-Rad Laboratories, Hercules, USA). The housekeeping gene GAPDH was used as endogenous control. Each sample and gene was conducted in triplicate. Primers (Ruibiotech, Beijing, China) used to amplify indicated genes were as following:

P21 forward: 5′-ATGAAATTCACCCCCTTTCC-3′

P21 reverse: 5′-CCCTAGGCTGTGCTCACTTC-3′

MDM2 forward: 5′-ATCTTGGCCAGTATATTATG-3′

MDM2 reverse: 5′-GTTCCTGTAGATCATGGTAT-3′

PUMA forward: 5′-GACGACCTCAACGCACAGTA-3′

PUMA reverse: 5′-AGGAGTCCCATGATGAGATTGT-3′

GAPDH forward: 5′-GGTCGTATTGGGCGCCTGGTC-3′

GAPDH reverse: 5′-GCCAGCATCGCCCCACTTGA-3′.

### Protein expression analysis

Western blot analysis was conducted by standard methods as previously described [51]. The relevant commercially primary antibodies were used to probe the alterations of protein as follows: Caspase-3, cleaved Caspase-3, p21 (Cell Signaling Technology, MA, USA), MDM2 (Abcam, Cambridge, UK), p53, PUMA, PARP (Santa Cruz Biotechnology, CA, USA). GAPDH or Actin (Beyotime, Shanghai, China) was used as a loading control. Anti-rabbit and anti-mouse IgG HRP-conjugated secondary antibodies from Santa Cruz Biotechnology, and antigen-antibody complexes were detected using Bio-Rad Clarity™ western ECL substrate (Bio-Rad Laboratory, CA, USA). In pharmacodynamic studies, protein expression analysis conducted as previously described.

### shRNA knockdown

The DePTC cells (TPC-1) were stably transfected with lentiviral vectors expressing shRNA-p53 corresponding to p53 nucleotides 611-629 [52] or scrambled sequence control shRNA (GenePharma, 20 Suzhou, China) for 3 days and then selected with 1.0 μg/ml puromycin (sigma, Shanghai, China) for 2 weeks.

### Immunohistochemical staining, immunofluorescence, *in situ* TUNEL staining

The xenograft tumor tissues were fixed and embedded in paraffin, cut at 4 μm. Cell proliferation ability was evaluated by scoring stained positive Tumor cells for Ki-67. Ki-67 antibody (Cell Signaling Technology, MA, USA) was 1:500 diluted and immunostaining was done following a standard method using DAB Substrate Kit (ZSGB-BIO, Beijing, China). Ki-67-positive cells were scored by visual detection in at least 500 cells using Leica microscope (Leica microsystem, Wetzlar, Germany). For expression of MDM2/p53 in tumor sections with APG115 treatment or vehicle, the protein level was measured by double-labeled *in situ* immunofluorescence as in previous methods [53]. Mouse p53 antibody (Santa Cruz Biotechnology, CA, USA) and rabbit MDM2 antibody (Abcam, Cambridge, UK) were used. TUNEL staining was performed with an *In Situ* Cell Death Detection Kit (Roche Diagnostics Corp., Mannheim, Germany). The stained slides were imaged and automatically digitized using panoramic MIDI (3D HSTECH, Budapest, Hungary), and then the data were analyzed using Panoramic Viewer Software (3D HSTECH). All positive cells were quantified by calculating in three random fields and the percentage of sum of positive cells was counted.

### APG115 pharmacokinetic and therapeutic efficacy in TPC-1 xenograft models

To develop xenograft tumors, 1×10^7^ TPC-1 cells suspended in 200 μl volume of 1:5 dilution of Matrigel (Corning, NY, USA) were injected subcutaneously into the right infra-axillary of female BABL/c nude mice. For pharmacodynamic studies, when tumors volume reached about 100~200 mm^3^, eight mice per group were gavaged with vehicle control (1% Klucel LF/ 0.1% Tween 80) or a single dose of the APG115 orally and sacrificed at the 0, 4, 8, 24, 48h post administration, with xenograft tumor tissue harvested for analyses. Tumor sizes and animal weights were recorded 2~3 times per week and the tumor volume was calculated as V (mm^3^) =1/2 × (length×width^2^). All animal experiments were carried out under the guide of Sun Yat-Sen University Committee for Use and Care of Laboratory Animals and approved by the animal experimentation ethics committee.

To detect apoptosis induction after APG115 treatment *in vivo*, bioluminescence images were collected, as previously described [44]. In brief, TPC-1 cells were transfected with H113 pLenti-CMV-GFP-linker-Luc-PGK-Puro lentiviral plasmid (Oobio, Shanghai, China). The culture medium was refreshed 48 h after infection, and the cells were then selected with 1 μg/mL puromycin (Life Technologies, Carlsbad, CA, USA) for two weeks to generate stable luciferase-expressing cells. On the indicated time-points after treatment, mice were intraperitoneally injected with Z-DEVD-aminoluciferin (VivoGlo Caspase-3/7 Substrate, Promega, Madison, USA) at a dose of 50 mg/kg in PBS, and then anesthetized with pentobarbital sodium (50 mg/kg, intraperitoneal injection). Mice were imaged on the IVIS Lumina XR imaging system (Caliper Life Sciences, MA, USA), taking images every 5 minutes for up to 25 minutes. Luciferase signal intensity was analyzed using Living Image software v.4.2 (Caliper Life Sciences, MA, USA).

### Statistical analysis

All experimental dates are reported as mean ± SD. *p-value < 0.05; **p-value < 0.01; ***p-value < 0.001; ****p-value < 0.0001. Significance was calculated using either two tailed paired or unpaired student's *t* test and ANOVA with Kruskal-Wallis test. All statistical analyses were performed using GraphPad Prism 6.0 (GraphPad Prism software Inc., San Diego, USA).

## SUPPLEMENTARY TABLE AND FIGURES



## Abbreviations

DePTC: dedifferentiated papillary thyroid cancer
DTC: differentiated thyroid cancer
PTC: papillary thyroid cancer
MDM2: murine double minute 2
RAI: radioactive iodine
TKIs: tyrosine kinase inhibitors
