# Supplementary material for: Restoration of p53 using the novel MDM2-p53 antagonist APG115 suppresses dedifferentiated papillary thyroid cancer cells
Source: Oncotarget. 2017 Apr 24;8(26):43008–22. doi: 10.18632/oncotarget.17398 (PMC5522123; doi:10.18632/oncotarget.17398)
Supplement: Supplementary file 1 [file oncotarget-08-43008-s001.pdf]

Restoration of p53 using the novel MDM2-p53 antagonist APG115 suppresses dedifferentiated papillary thyroid cancer cells

SUPPLEMENTARY MATERIALS

SUPPLEMENTARY TABLE AND FIGURES

Supplementary Table 1: IC_{50} of APG115 and its analogue SAR405838 in DeTPC cells with different p53 Status

Cell lines	p53 Status	APG115 IC_{50} (nM)	SAR405838 IC_{50} (nM)
KTC-1	Wild-type	94.8 ± 38.0	276.6 ± 42.3
TPC-1	Wild-type	133.4 ± 28.3	576.3 ± 17.5
TPC-1 shNC	Wild-type	158.2 ± 30.3	__
TPC-1 shp53	Deletion	>10000	__
B-CPAP	Mutation	>10000	__

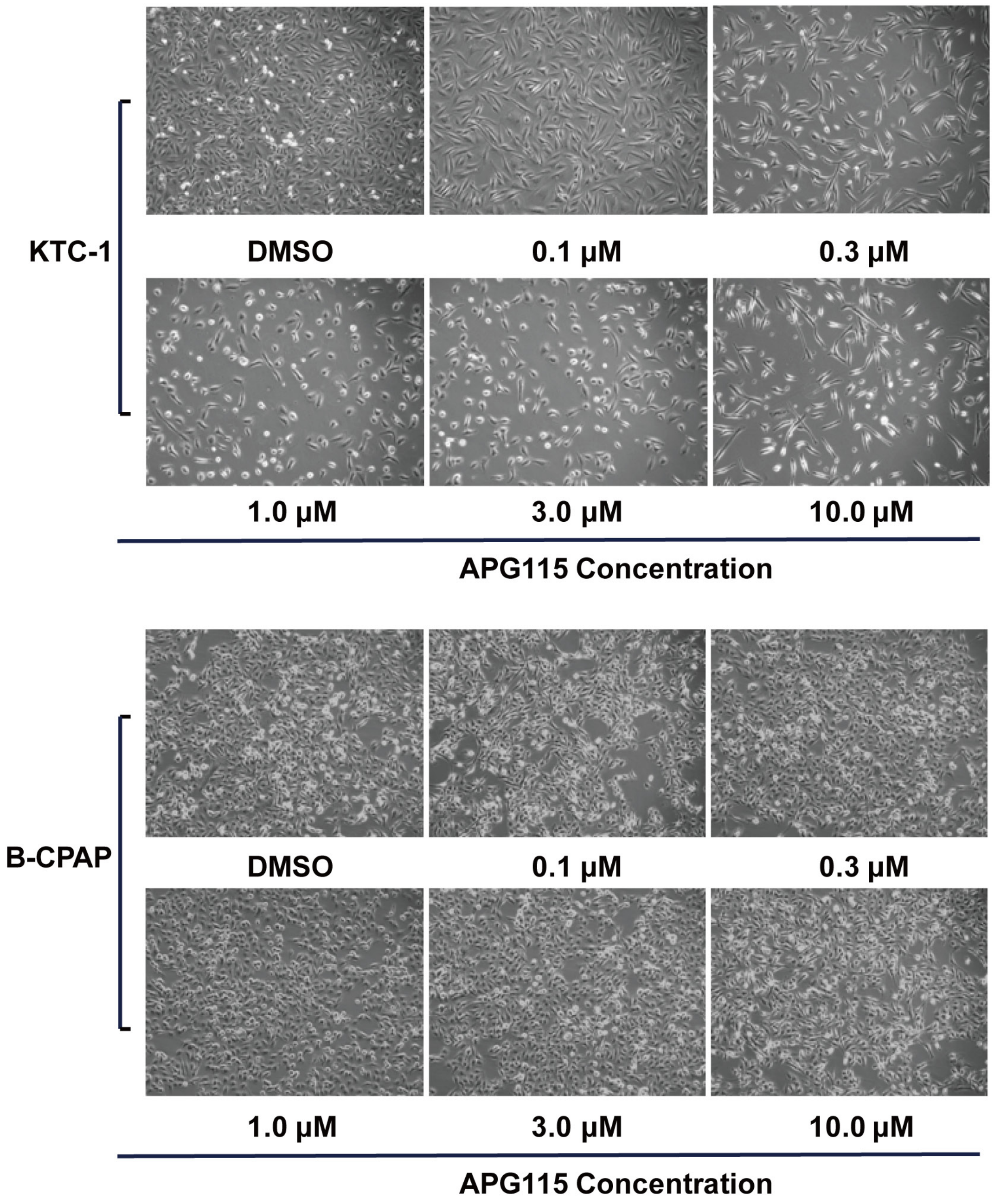

Supplementary Figure 1: Cell morphology profiles changing in response to APG115 treatment. The morphology profile of KTC-1 cells changed in response to incubation with the indicated concentrations of APG115 for 72 h, but that of B-CPAP did not.

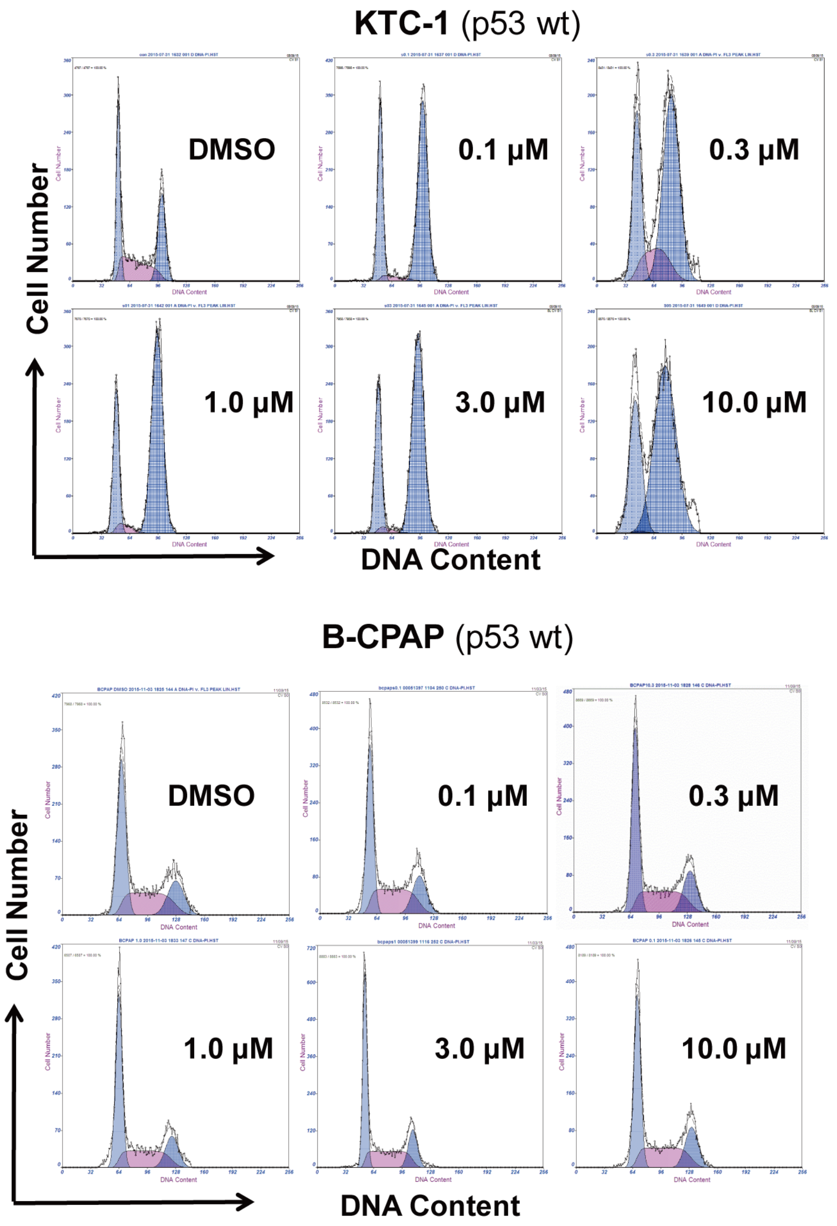

Supplementary Figure 2: APG115 elicited cell cycle arrest in a p53-dependent manner. Incubation with APG115 induced a concentration-dependent cell cycle arrest in G2/M phases and a reduction in the number of cells in the S-phase in TPC-1 and KTC-1 cells retaining wild-type p53, but not in B-CPAP cells with mutated p53.
